# Hypoxia-activated prodrug TH-302 decreased survival rate of canine lymphoma cells under hypoxic condition

**DOI:** 10.1371/journal.pone.0177305

**Published:** 2017-05-10

**Authors:** Hiroki Yamazaki, Yu-Chang Lai, Morihiro Tateno, Asuka Setoguchi, Yuko Goto-Koshino, Yasuyuki Endo, Munekazu Nakaichi, Hajime Tsujimoto, Naoki Miura

**Affiliations:** 1 Veterinary Teaching Hospital, Joint Faculty of Veterinary Medicine, Kagoshima University, Korimoto, Kagoshima, Japan; 2 Laboratory of Veterinary Internal Medicine, Joint Faculty of Veterinary Medicine, Kagoshima University, Korimoto, Kagoshima, Japan; 3 Bayside Animal Clinic, Yokohama, Kanagawa, Japan; 4 Department of Veterinary Internal Medicine, Graduate School of Agricultural and Life Sciences, The University of Tokyo, Bunkyo, Tokyo, Japan; 5 Laboratory of Veterinary Radiology, Department of Veterinary Medicine, Joint Faculty of Veterinary Medicine, Yamaguchi University, Yoshida, Yamaguchi, Japan; University of Nebraska Medical Center, UNITED STATES

## Abstract

We tested the hypotheses that hypoxic stimulation enhances growth potentials of canine lymphoma cells by activating hypoxia-inducible factor 1α (HIF-1α), and that the hypoxia-activated prodrug (TH-302) inhibits growth potentials in the cells. We investigated how hypoxic culture affects the growth rate, chemoresistance, and invasiveness of canine lymphoma cells and doxorubicin (DOX)-resistant lymphoma cells, and influences of TH-302 on survival rate of the cells under hypoxic conditions. Our results demonstrated that hypoxic culture upregulated the expression of HIF-1α and its target genes, including *ATP-binding cassette transporter B1* (*ABCB1*), *ATP-binding cassette transporter G2* (*ABCG2*), *platelet-derived growth factor* (*PDGF*), *vascular endothelial growth factor* (*VEGF*), and *survivin*, and enhanced the growth rate, DOX resistance, and invasiveness of the cells. Additionally, TH-302 decreased the survival rate of the cells under hypoxic condition. Our studies suggest that hypoxic stimulation may advance the tumorigenicity of canine lymphoma cells, favoring malignant transformation. Therefore, the data presented may contribute to the development of TH-302-based hypoxia-targeting therapies for canine lymphoma.

## Introduction

Canine hematopoietic malignancies have been widely investigated and their pathological mechanisms studied to develop therapeutic strategies for human patients, and lymphoma in dogs may be useful as a model of human disease [1]. Lymphoma occurs in humans in two main forms, Hodgkin and non-Hodgkin lymphoma (NHL). NHL is the most common lymphoma, originating from B or T cells [2]. Canine lymphomas and NHL are almost indistinguishable because of the striking similarities in their biology, pathology, gene expression patterns, and immunological features [1–3]. Both tumors also display similar therapeutic responses and clinical courses. Although multidrug chemotherapy based on doxorubicin (DOX) and DNA-alkylating agents is highly efficacious against these tumors, multidrug resistance ultimately leads to treatment failure, resulting in poor outcomes [1–3]. Therefore, a novel therapeutic approach is required to combat canine lymphoma and NHL.

Hypoxia-inducible factor 1α (HIF-1α) is a transcription factor that is activated in response to oxygen deficiency, and HIF-1α expression is activated in several cancers under the intratumoral hypoxic stress that arises during pathogenic processes [4, 5]. HIF-1α is expressed at high levels in canine lymphoma and NHL, whereas it is typically expressed at low or negligible levels in normal tissues under normoxia [5–7]. HIF-1α activity leads to the upregulation of target genes, which advance cancer progression, angiogenesis, cell survival, and cell invasion [4]. Currently, more than 70 putative HIF-1α target genes have been identified, including ATP-binding cassette transporters B1 (*ABCB1*), ATP-binding cassette transporters G2 (*ABCG2*), vascular endothelial growth factor (*VEGF*), platelet-derived growth factor (*PDGF*), and *survivin* [4, 8, 9]. Therefore, determining the relationship between HIF-1α activation and the survival potential of canine lymphoma cells under hypoxic conditions may provide insight into tumorigenesis and provide medically valuable information for the treatment of both NHL and canine lymphoma.

The 2-nitroimidazole moiety of the hypoxia-activated prodrug evofosfamide (TH-302) is triggered by hypoxia to release the DNA-alkylating moiety dibromo isophosphoramide mustard (Br-IPM) within the hypoxic regions of tumors [10–12]. TH-302 is thought to function as a hypoxia-targeting drug in human cancer patients, including those with leukemia, pancreatic cancer, or soft-tissue sarcoma [10–13]. Phase II clinical trials in which TH-302 was used to treat patients with pancreatic cancer or soft-tissue sarcoma were deemed successful [10–12]. A combination of TH-302 and DOX or gemcitabine has also been tested, and in a phase II trial of this combination in patients with pancreatic cancer, progression-free survival (PFS) was significantly longer when TH-302 was administered with gemcitabine than when gemcitabine was administered alone [10]. However, whether TH-302 can be used to treat canine and human lymphoma is unknown. Testing its effects *in vitro* may contribute to improvement of the treatment.

In this study, our aim was to investigate how hypoxic culture influences growth rate, chemoresistance, and invasiveness of canine lymphoma cells, and influences of TH-302 on survival rate of the cells under hypoxic conditions. We hypothesized that HIF-1α is activated in the hypoxic environment formed during the proliferation of the cells, and that TH-302 induces inhibitory activities for the cell survival.

## Materials and methods

### Cell lines and cultures

Canine lymphoma cells (CL-1 and GL-1) [14, 15], DOX-resistant lymphoma cells (CL-1DR and GL-1DR) and mononuclear cells were used in this study. The CL-1DR and GL-1DR were generated from the corresponding parental cells (CL-1 and GL-1) with a previously reported procedure [16], the details of which are given in the S1 File and S1 Table. Mononuclear cells were isolated from the fresh peripheral blood of a healthy 1-year-old, intact female beagle by a specific gravity centrifugal method using LymphoPrep (Cosmo Bio, Tokyo, Japan). All cells were cultured in RPMI 1640 (Gibco, Grand Island, NY, USA) supplemented with 10% heat-inactivated fetal bovine serum (FBS; Cosmo Bio, Tokyo, Japan) and 1% L-glutamine (BioWhittaker, Walkersville, MD, USA) under various O_2_ concentrations (21%, 10%, 5%, or 1% O_2_) with 5% CO_2_ at 37°C in a tri-gas incubator (HERAcell® 150i; Thermo Scientific, Waltham, MA, USA).

### Reagents

Cells were treated with various concentrations of DOX (Sigma, St Louis, MO, USA) or TH-302 (Threshold Pharmaceuticals, South San Francisco, CA, USA) dissolved and diluted in 0.01% dimethyl sulfoxide.

### Quantitative real-time reverse transcription polymerase chain reaction (qRT-PCR)

After culture for 24 h under normoxic (21% O_2_) or hypoxic conditions (10%, 5%, and 1% O_2_), the mRNA expression of *HIF-1α*, *ABCB1*, *ABCG2*, *PDGF*, *VEGF*, and *survivin* in the cells was evaluated with qRT-PCR. After treatment with 50 μM TH-302 for 12 h during culture under 21% and 5% O_2_, the expression of *HIF-1α* was evaluated. The details included in the S1 File, S2 Table and S1, S2, S3 and S4 Figs.

### Western blotting

After the cells were cultured for 12 h under normoxia (21% O_2_) or hypoxia (5% or 1% O_2_) and then treated for 12 h with 50 μM TH-302 under 21% or 5% O_2_, the total, nuclear and cytoplasmic proteins were extracted from them with the Nuclear/Cytosolic Fraction Kit (Cell Biolabs, San Diego, CA, USA). The details included in the S1 File and S5 Fig.

### Cell viability assay

In a pilot study, cell viability was evaluated after culture under 21%, 10%, 5%, or 1% O_2_ for 0, 24, 48, 72, or 96 h, with the Cell Proliferation Kit I (Roche, Indianapolis, IN, USA), according to the manufacturer’s instructions. The optical density (OD) of each well was measured at a wavelength of 570 nm (OD_570_) using an iMark microplate spectrophotometer (Bio-Rad, Hercules, CA, USA). Cell viability was determined as (OD_570_ treated cells/OD_570_ untreated cells at time 0) × 100. A long-term culture of cells under 5% O_2_ was then established based on the pilot study data (S2 Fig). At 30 and 90 days after culture under 21% or 5% O_2_, cell viability was evaluated as previously described.

### Drug sensitivity testing

The DOX sensitivity of cells was evaluated after their long-term culture under hypoxic conditions. After 30 or 90 days in culture under 21% or 5% O_2_, the cells were left untreated or treated with four different concentrations of DOX (1, 10, 100, or 1000 nM). After treatment for 0, 24, 48, or 72 h, cell viability was evaluated as previously described. TH-302 sensitivity was evaluated after the cells were cultured under various O_2_ concentrations. After treatment with various concentrations of TH-302 (0, 20, 40, 60, 80, or 100 μM) for 24 h under 21%, 10%, 5%, or 1% O_2_, the cell viability was evaluated as previously described.

### Cell invasion assay

The cell invasive capacity was assessed with the CytoSelect 24-Well Cell Invasion Assay kit (Cell Biolabs, San Diego, CA, USA), according to the manufacturer’s instructions. Briefly, after 90 days in culture under 21% or 5% O_2_, the cells were harvested, washed with phosphate-buffered saline, and suspended in serum-free medium at 5 × 10^6^ cells/mL. Medium (500 μL) containing 10% FBS was added to the lower well of the invasion plate, and 300 μL of the cell suspension was added to the inside of each insert. The plates were incubated at 37°C in 5% CO_2_ and 21% or 5% O_2_ for 48 h. The interiors of the inserts were gently swabbed to remove noninvasive cells. The invasive cells on the lower surfaces of the filters were stained and observed with light microscopy. The invasive cells were collected, and the percentage of cell invasion was quantified with the Cell Proliferation Kit I, as described above.

### Assessment of apoptosis

Apoptosis was evaluated with the Annexin V–Biotin Apoptosis Detection Kit (with streptavidin-FITC; Blue Heron Biotechnology, Bothell, WA, USA), according to the manufacturer’s instructions. After treatment with 50 μM TH-302 for 24 h under 21% or 5% O_2_, the cells were processed for annexin V staining. The percentage of apoptotic cells was quantified under a fluorescence microscope (BioRevo BZ-9000; Keyence Corp., Osaka, Japan) and observed with light microscopy after Wright–Giemsa staining.

### Statistical analysis

Statistical analyses were performed with standard software (SPSS Inc., Chicago, IL USA). The data were analyzed with Dunnett’s test or one-way analysis of variance followed by the Tukey post hoc test. Quantitative values are expressed as the means ± standard deviations (SD) of three separate experiments, and *P* values of less than 0.05 are considered significant.

## Results

### Hypoxia enhanced expression of nuclear HIF-1α and its target gene in lymphoma cells

**Fig 1:**
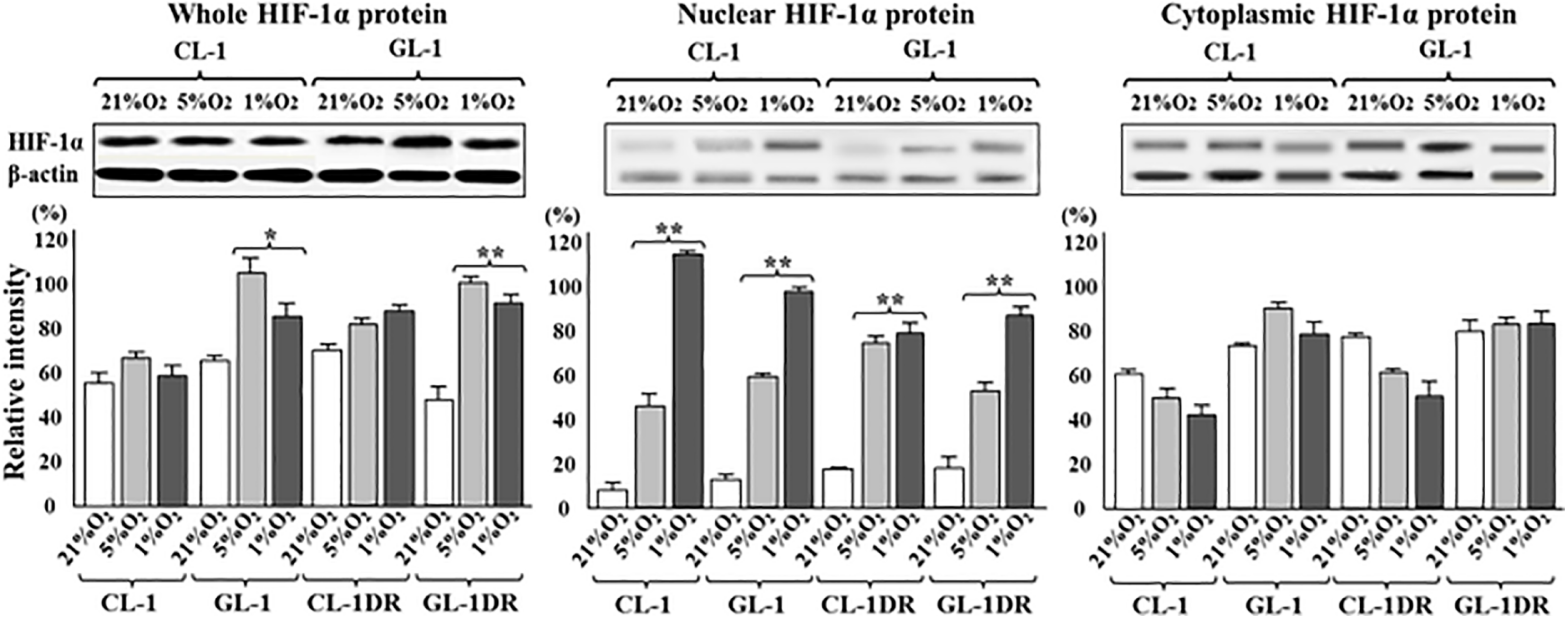
Total, nuclear and cytosolic localization of HIF-1α protein in lymphoma cells after hypoxic culture. After 12 h in culture under 21%, 5%, or 1% O_2_, total, nuclear and cytosolic HIF-1α protein was detected with a western blotting analysis. Immunoreactive bands were quantified and are presented as relative intensities (%) normalized to those of β-actin. Each bar represents the mean ± SD of three separate experiments. **P < 0.01 vs control (Dunnett’s test).

After the cells were cultured for 12 h under 5% or 1% O_2_, the expression of the nuclear protein HIF-1α was upregulated in the CL-1, CL-1DR, GL-1, and GL-1DR. The relative intensities of the immunoreactive bands were significantly greater than those of the controls (21% O_2_; Fig 1). The expression of the *HIF-1α*, *ABCB1*, *ABCG2*, *VEGF*, *PDGF*, and *survivin* genes was significantly higher in CL-1, GL-1, CL-1DR, and GL-1DR after culture for 24 h under 5% or 1% O_2_ than in the cells cultured under 10% O_2_ (S3 Fig). However, the expression of most genes did not differ significantly between the cells cultured under 1% O_2_ and those cultured under 5% O_2_. These data suggest that hypoxic culture increases HIF-1α protein expression in the nucleus, and enhance expression of the target genes.

### Hypoxia enhanced the growth rate, chemoresistance, and invasiveness of lymphoma cells

**Fig 2:**
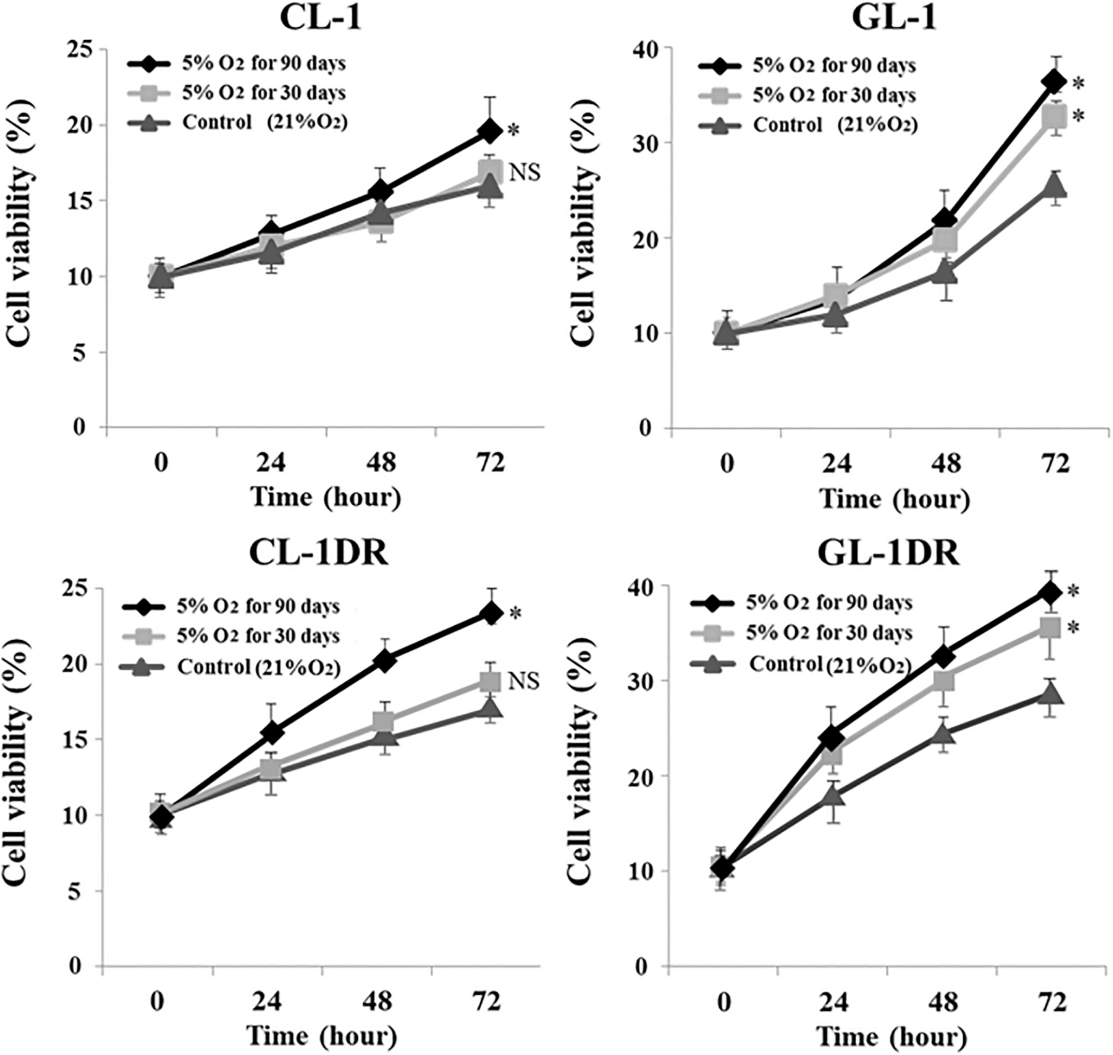
Viability of lymphoma cells after hypoxic culture. Cell viability was evaluated after 30 or 90 days in hypoxic culture (5% O_2_). Relative cell viability is presented as a percentage (%) of the control value. Each bar represents a mean ± SD. *P < 0.05; NS, not significant vs control (post hoc test).

**Fig 3:**
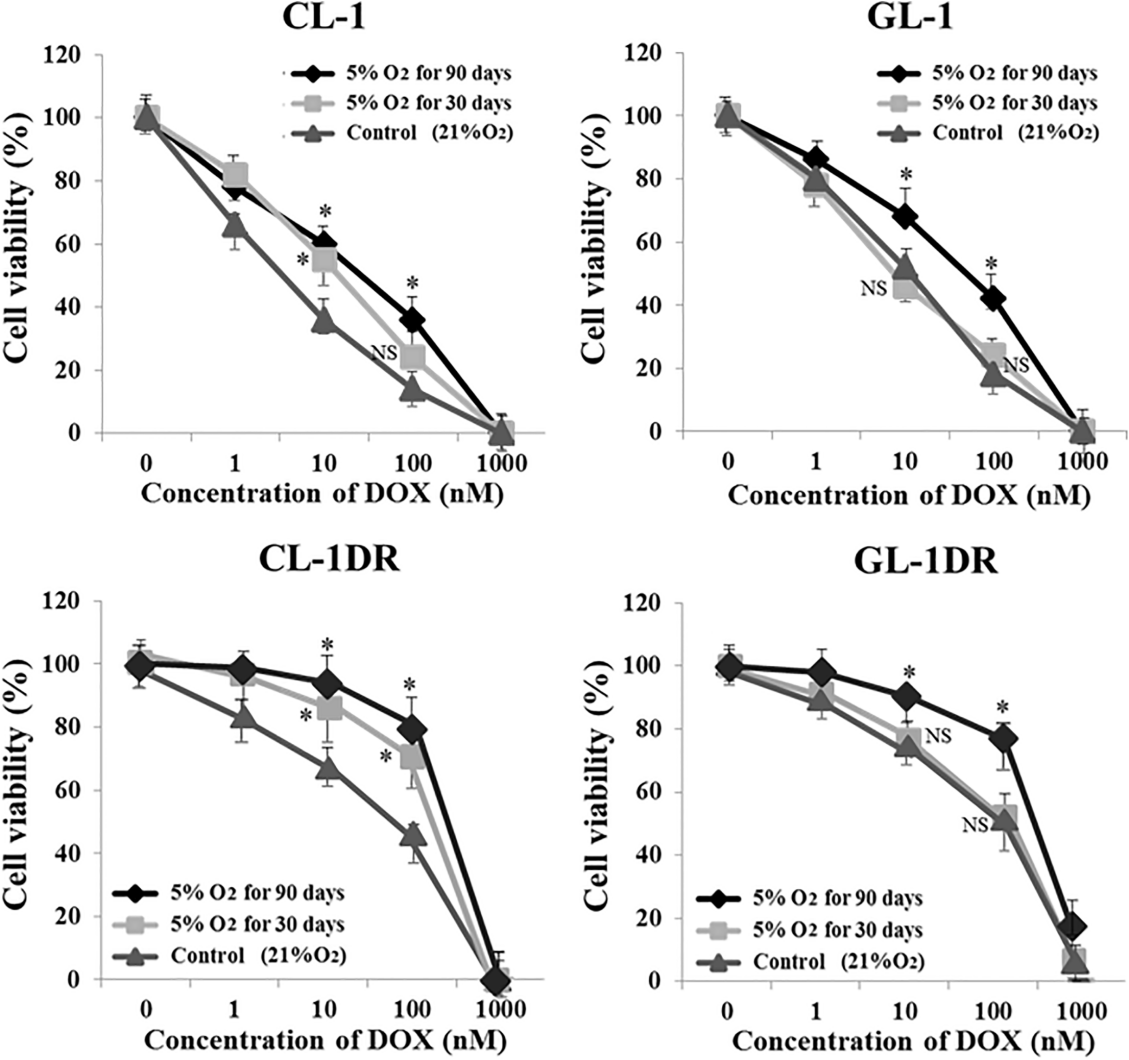
Sensitivity of lymphoma cells to doxorubicin (DOX) after hypoxic culture. DOX sensitivity was evaluated in lymphoma cells after 30 and 90 days in hypoxic culture (5% O_2_). Relative cell viability is shown as a percentage (%) of the control value, and each bar represents a mean ± SD. *P < 0.05; NS, not significant vs control (Dunnett’s test).

**Fig 4:**
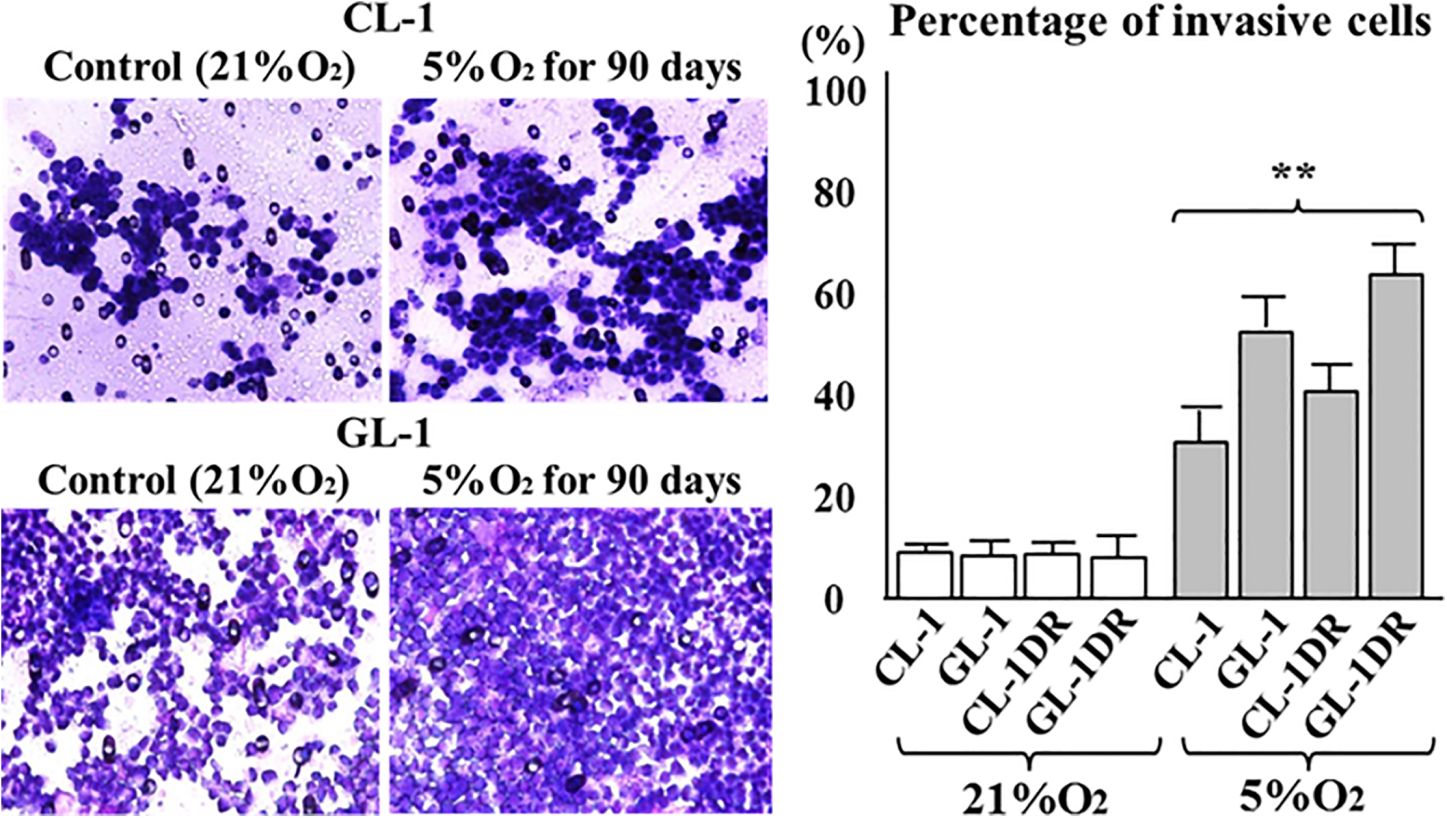
Invasive capacity of lymphoma cells after hypoxic culture. After 90 days in hypoxic culture (5% O_2_), the percentages of invasive cells were evaluated. Data are presented as percentages (%) of the control values, and each bar represents the mean ± SD of three separate experiments. **P < 0.01 vs control (Dunnett’s test).

After culture for 90 days under 5% O_2_, the viability of CL-1 and CL-1DR was significantly higher than that of the controls (21% O_2_; Fig 2). After 30 or 90 days in culture under 5% O_2_, the viability of GL-1 and GL-1DR was significantly higher than that of the controls (Fig 2). After 30 or 90 days in culture under 5% O_2_, the sensitivity of CL-1 and CL-1DR to DOX was significantly lower than that of the controls (21% O_2_; Fig 3). After 90 days in culture under 5% O_2_, the sensitivity of GL-1 and GL-1DR to DOX was also significantly lower than that of the controls (Fig 3). After 90 days in culture under 5% O_2_, the percentages of invasive CL-1, CL-1DR, GL-1, and GL-1DR were significantly higher than those of the controls (21% O_2_; Fig 4). These data suggest that long-term exposure to hypoxia induces cell transformation, and enhances the growth rate, chemoresistance, and invasive capacity or migration of cells.

### TH-302 decreased survival rate of lymphoma cells under hypoxia

**Fig 5:**
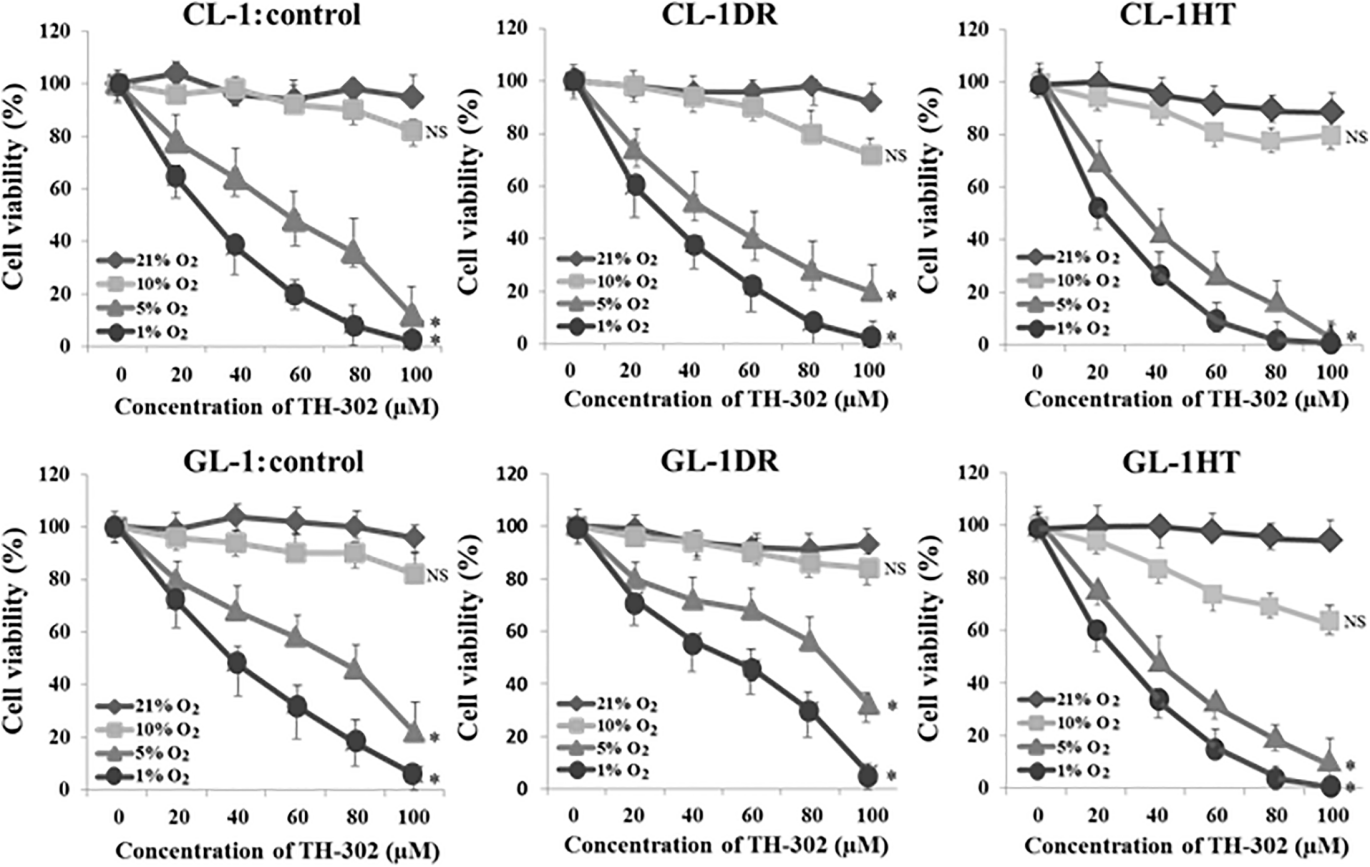
Sensitivity of lymphoma cells to TH-302 after hypoxic culture. Cell viability was determined after treatment with various concentrations of TH-302 for 24 h during culture under 21%, 10%, 5%, or 1% O_2_. Relative cell viability is presented as a percentage (%) of the control value. Each bar represents a mean ± SD. *P < 0.05; NS, not significant vs control (Dunnett’s test).

**Fig 6:**
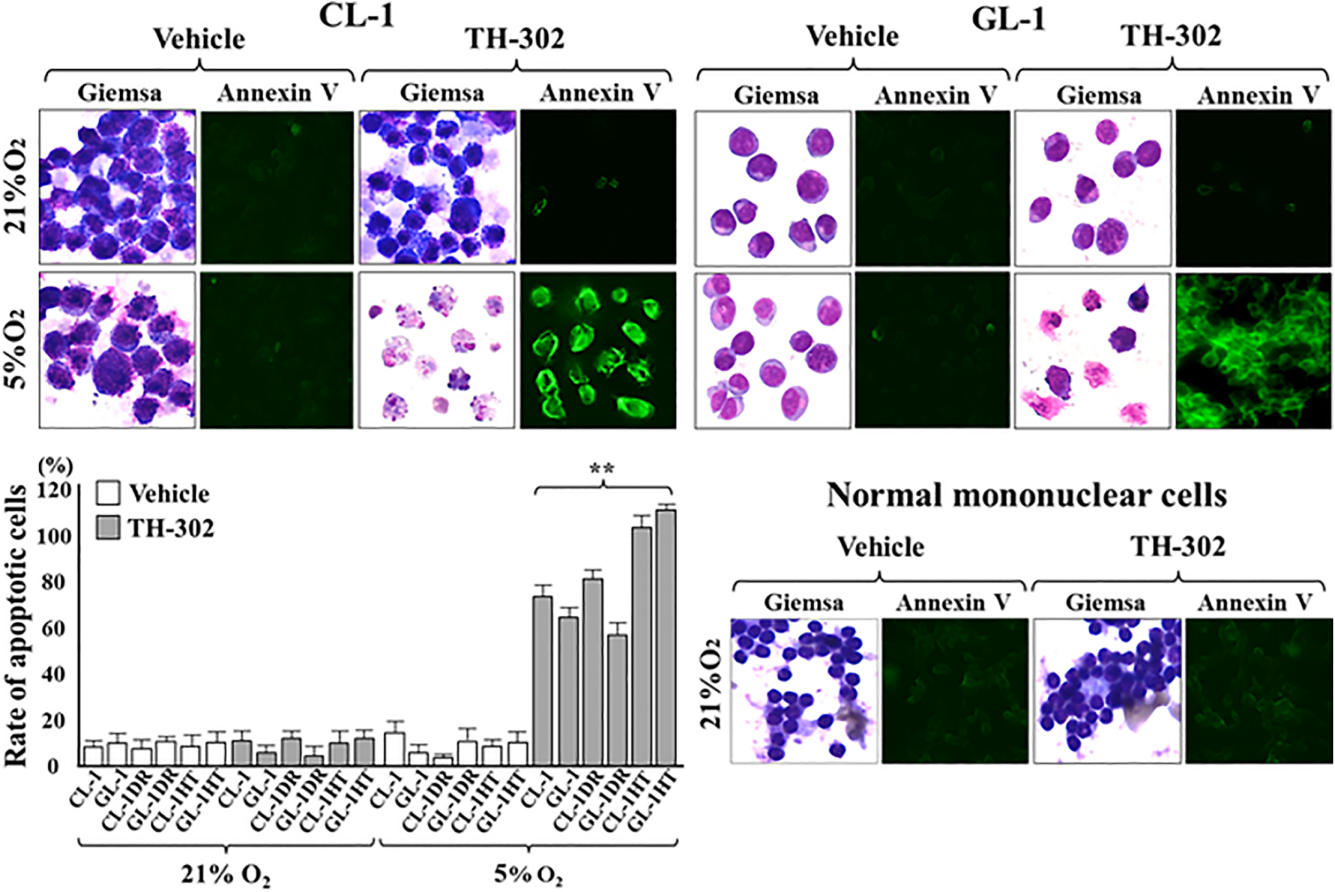
Apoptosis in lymphoma cells after treatment with TH-302. After treatment with 50 μM TH-302 for 24 h during culture under 5% O_2_, apoptotic cells were evaluated with fluorescent annexin V staining (400× magnification). Light microscope images were obtained after Wright–Giemsa staining (1000× magnification). The percentage of apoptotic cells was quantified with a fluorescent image analyzer, and the data are presented as percentages (%) of the control values. Each bar represents the mean ± SD of three separate experiments. **P < 0.01 (Dunnett’s test).

After treatment with 50 μM TH-302 for 24 h under 5% or 1% O_2_, the viability of CL-1, CL-1DR, GL-1, GL-1DR, CL-1HT (the hypoxia tolerance cells cultured under 5% O_2_ for 90 days), and GL-1HT was significantly lower than that of the controls (21% O_2_; Fig 5). However, after treatment with 50 μM TH-302 for 24 h under 10% O_2_, there were no significant differences in the viability of any cells (Fig 5). After treatment with 50 μM TH-302 for 24 h under 5% O_2_, the percentages of apoptotic cells, including CL-1, CL-1DR, GL-1, GL-1DR, CL-1HT, and GL-1HT, were significantly higher than those of the vehicle-treated cells, and nuclear fragmentation and sequential decay were observed in these cells after Wright–Giemsa staining (Fig 6). After treatment with 50 μM TH-302 for 24 h under 21% O_2_, the percentages of apoptotic cells in all the cell types were not significantly different from those of the vehicle-treated cells, and there were negligible morphological abnormalities among the cells (Fig 6). These data demonstrate that the growth inhibitory effects of TH-302 are triggered by hypoxic conditions (≤5% O_2_). After treatment with TH-302 for 24 h under 21% O_2_, there were negligible morphological abnormalities in the mononuclear cells, and the percentage of apoptotic cells did not differ significantly from that in the vehicle-treated control cells (Fig 6). These data suggest that TH-302 decreases the survival rate of canine lymphoma cells under hypoxia, however exerts few adverse effects on lymphocytes and monocytes in normally oxygenated blood.

After the cells were treated with 50 μM TH-302 for 12 h under 5% O_2_, *HIF-1α* gene expression in the CL-1, CL-1DR, GL-1, GL-1DR. CL-1HT, and GL-1HT under 5% O_2_ were significantly lower than those from the vehicle-treated cells (S4 Fig). Additionally, the nuclear and cytosolic levels of HIF-1α protein in the TH-302-treated cells were downregulated, and the relative intensities of the immunoreactive bands on western blots were significantly lower than those from the vehicle-treated cells (S5 Fig).

## Discussion

Our results demonstrated that hypoxic culture upregulated expression of HIF-1α and its target genes, including *ABCB1*, *ABCG2*, *PDGF*, *VEGF*, and *survivin*, in canine lymphoma cells, and enhanced growth rate, chemoresistance and invasive capacity. Angiogenesis and resistance to apoptosis are required to confer a survival advantage on cancer cells, and the overexpression of PDGF, VEGF and survivin promotes cell proliferation, cell migration and apoptotic resistance [17–19]. Several research groups have reported that elevated HIF-1α levels have been linked to poor prognoses in human diffuse large-B-cell lymphoma [20, 21]. Other groups suggested that the expression patterns of PDGF, VEGF and survivin in canine lymphoma correlate with clinical stage and histological grade [19, 22, 23]. Hypoxic stimulation effectively may improve growth potential and invasiveness of canine lymphoma cells in response to upregulation of *PDGF*, *VEGF* and *survivin* expression. High-grade lymphomas in dogs and humans are commonly accompanied by high levels of serum lactate dehydrogenase and thymidine kinase, implying that cell proliferation is accelerated by oxygen deficiency [24, 25]. Our data showed that long-term culture under 5% O_2_ promoted the growth of canine lymphoma cells, whereas exposure to 1% O_2_ inhibited their growth. Severe hypoxia (≤1% O_2_) may induce cytotoxicity or apoptosis on lymphomas [26]. Conversely, if a mildly hypoxic or microaerobic environment is required for tumor development, therapies that target mild hypoxia may be useful for treating lymphomas.

It has generally been thought that lymphomas in humans and dogs acquire multidrug resistance from the continuous activation of drug transporters, such as ABCB1 and ABCG2, after long-term exposure to anticancer drugs, inducing chemical tolerance in the patient [3, 27]. Interestingly, we have shown here that mild hypoxic stimulation also reduced the sensitivity to DOX of DOX-resistant lymphoma cells generated with conventional methods. This evidence reinforces the novel hypothesis that HIF-1α plays a major role in the chemoresistance mechanism or promotes the activation of drug transporters in lymphoma cells [28]. Therefore, the inhibition of HIF-1α may abolish chemoresistance and restore the effects of anticancer drugs.

Our results demonstrated that TH-302 significantly decreased survival rate of canine lymphoma cells under hypoxic conditions (≤5% O_2_), and downregulated HIF-1α gene and protein. However, it has not been recognized until now how TH-302 downregulates HIF-1α expression [29]. The total amount of Br-IPM released as a DNA-alkylating moiety is determined by the oxygen concentration [12]. One research group reported that TH-302 showed cytotoxic effects in multiple myeloma cells at oxygen concentrations of <1.5% O_2_ or at <10 mmHg partial O_2_ pressure [29]. Canine lymphoma and NHL are generally highly sensitive to DNA-alkylating agents [30, 31]. Therefore, even small amounts of Br-IPM released under mildly hypoxic conditions may damage the cells by DNA alkylation. Some lymphoma cells can utilize the aerobic glycolytic system to obtain a growth advantage [32]. At or near the growth-plateau phase of these cells, intracellular oxygen levels are thought to be reduced by the imbalance in oxygen consumption and supply [33]. This suggests that the effects of TH-302 may be accelerated during rapid cell growth.

Our results demonstrate that TH-302 exerts few adverse effects on canine lymphocytes and monocytes under normoxia. In a phase I study of the use of TH-302, the most common adverse events were nausea, skin rash, and fatigue, whereas hematological toxicity was mild and limited [13, 34, 35]. A combination of TH-302 and conventional drugs is expected to enhance the antitumor effects without marked adverse events [35, 36]. When TH-302 was used in combination with DOX, it displayed a median overall survival time of 6 months and a median PFS of 21.5 months in patients with advanced soft-tissue sarcoma, with no evidence of severe toxicity and a clinical benefit rate of 84% (complete response 2%, partial response 34%, stable disease 48%, and disease progression 16%) [10, 36]. Multidrug therapy is commonly useful for lymphoma in humans and dogs, and a treatment protocol including TH-302 may improve their clinical outcomes.

Our findings showed that hypoxic culture upregulated the expression of HIF-1α and its target genes in canine lymphoma cells, and enhanced their growth rate, DOX resistance, and invasiveness. However, TH-302 decreased survival rate of the cells under hypoxic conditions. Our study suggests that hypoxic stimulation enhances the tumorigenicity of canine lymphoma, favoring malignant transformation. These data presented here may contribute to the basic assessment of TH-302-based hypoxia-targeting therapies for lymphomas. Further translational research is required to evaluate the safety of TH-302 and to determine whether its combination with conventional anticancer drugs enhances its effects.

## Supporting information

S1 Fig Relative expression of the candidate internal reference genes. The mRNA expression, including β-actin (*ACTB*), glyceraldehyde-3-phosphate dehydrogenase (*GAPDH*), hypoxanthine phosphoribosyltransferase 1 (*HPRT*), ribosomal protein L13a (*RPL13A*), and TATA box-binding protein (*TBP*) were analyzed with qRT-PCR for selection of adequate internal reference gene. After 24 h in hypoxic culture (10%, 5% and 1% O_2_), the expression levels of these five genes in the CL-1 and GL-1 was quantified with the geNorm software (version 3.5). All expression was normalized to that of the control samples (21% O_2_), and each bar represents a mean ± SD. *P < 0.05 and **P < 0.01 (Dunnett’s test).
(TIF)

S2 Fig The viability of CL-1 and GL-1 cells after 0, 24, 48, 72, or 96 h in culture under 21%, 10%, 5%, and 1% O_2_. Relative cell viability is presented as a percentage (%) of the control value, and each bar represents a mean ± SD. **P < 0.01 vs the control cultured under 21% O_2_ (post hoc test). When cultured for 96 h under 1% O_2_, cell viability was significantly lower than that of the control cells (21% O_2_), whereas cell viability did not differ significantly under mildly hypoxic conditions (10% or 5% O_2_).
(TIF)

S3 Fig Relative mRNA expression in lymphoma cells after hypoxic culture. After 24 h in hypoxic culture (10%, 5%, or 1% O_2_), the mRNA expression of hypoxia-inducible factor 1α (*HIF-1α*), ATP-binding cassette transporter B1 (*ABCB1*), ATP-binding cassette transporter G2 (*ABCG2*), endothelial growth factor (*VEGF*), platelet-derived growth factor (*PDGF*), and *survivin* was analyzed with qRT-PCR. All expression was normalized to that of the control samples (21% O_2_), and each bar represents a mean ± SD. *P < 0.05 and **P < 0.01 (Dunnett’s test).
(TIF)

S4 Fig Relative mRNA expression of *HIF-1α* in lymphoma cells after treatment with vehicle and TH-302. After treatment with 50 μM TH-302 for 12 h during culture under 5% O_2_, the mRNA expression was analyzed with qRT-PCR. All expression was normalized to that of the control samples (21% O_2_), and each bar represents a mean ± SD. *P < 0.05 and **P < 0.01 (Dunnett’s test).
(TIF)

S5 Fig Nuclear and cytosolic localization of HIF-1α protein in lymphoma cells after treatment with TH-302. After treatment with 50 μM TH-302 for 12 h during culture under 5% O_2_, the nuclear and cytosolic localization of HIF-1α was detected with western blotting. Immunoreactive band intensities are presented as percentages (%) of the control values. Each bar represents the mean ± SD of three separate experiments. **P < 0.01 vs control (Dunnett’s test).
(TIF)

S1 Table Baseline information on the canine lymphoma cells used in the study. (DOCX)

S2 Table Target primer sequences used in this study. (DOCX)

S1 File Supporting methods in this study. (DOCX)

## References

[1] Ito D, Frantz AM, Modiano JF. Canine lymphoma as a comparative model for human non-Hodgkin lymphoma: recent progress and applications.Vet Immunol Immunopathol. 2014;159: 192–201. doi: 10.1016/j.vetimm.2014.02.016 24642290 10.1016/j.vetimm.2014.02.016 PMC4994713

[2] Perry AM, Diebold J, Nathwani BN, MacLennan KA, Müller-Hermelink HK, Bast M. Non-Hodgkin lymphoma in the developing world: review of 4539 cases from the International Non-Hodgkin Lymphoma Classification Project. Haematologica. 2016;e: 148809. 10.3324/haematol.2016.148809 PMC5046654 27354024

[3] Zandvliet M. Canine lymphoma: a review. Vet Q. 2016;36: 76–104. doi: 10.1080/01652176.2016.1152633 26953614 10.1080/01652176.2016.1152633

[4] Xia Y., Choi H.K., Lee K., Recent advances in hypoxia-inducible factor (HIF)-1 inhibitors, Eur. J Med Chem. 49 (2012) 24–40. doi: 10.1016/j.ejmech.2012.01.033 22305612 10.1016/j.ejmech.2012.01.033

[5] Talks KL, Turley H, Gatter KC, Maxwell PH, Pugh CW, Ratcliffe PJ, et al The expression and distribution of the hypoxia-inducible factors HIF-1alpha and HIF-2alpha in normal human tissues, cancers, and tumor-associated macrophages, Am. J Pathol. 2000;157: 411–421. 10934146 10.1016/s0002-9440(10)64554-3 PMC1850121

[6] Hernandez-Luna MA, Rocha-Zavaleta L, Vega MI, Huerta-Yepez S. Hypoxia inducible factor-1α induces chemoresistance phenotype in non-Hodgkin lymphoma cell line via up-regulation of Bcl-xL. Leuk Lymphoma. 2013;54: 1048–1055. doi: 10.3109/10428194.2012.733874 23013270 10.3109/10428194.2012.733874

[7] Kambayashi S, Igase M, Kobayashi K, Kimura A, Shimokawa-Miyama T, Baba K, et al Hypoxia inducible factor 1α expression and effects of its inhibitors in canine lymphoma. J Vet Med Sci. 2015;77: 1405–1412. doi: 10.1292/jvms.15-0258 26050843 10.1292/jvms.15-0258 PMC4667657

[8] Bos R, van Diest PJ, de Jong JS, van der Groep P, van der Valk P, van der Wall E. Hypoxia-inducible factor-1alpha is associated with angiogenesis, and expression of bFGF, PDGF-BB, and EGFR in invasive breast cancer. Histopathology. 2005;46: 31–36. doi: 10.1111/j.1365-2559.2005.02045.x 15656883 10.1111/j.1365-2559.2005.02045.x

[9] Chen YQ, Zhao CL, Li W. Effect of hypoxia-inducible factor-1alpha on transcription of survivin in non-small cell lung cancer. J Exp Clin Cancer Res. 2009;28: 29 doi: 10.1186/1756-9966-28-29 19245702 10.1186/1756-9966-28-29 PMC2663545

[10] Hendifar AE, Tine BV, Chawla N, Yafee P, Chawla SP. A novel approach to soft tissue sarcoma therapy: targeting tumor hypoxia. Ann Cancer Res. 2015;2: 5.

[11] Garrido-Laguna I, Hidalgo M. Pancreatic cancer: from state-of-the-art treatments to promising novel therapies. Nat Rev Clin Oncol. 2015;12: 319–334. doi: 10.1038/nrclinonc.2015.53 25824606 10.1038/nrclinonc.2015.53

[12] Phillips RM. Targeting the hypoxic fraction of tumours using hypoxia-activated prodrugs. Cancer Chemother Pharmacol. 2016;77: 441–457. doi: 10.1007/s00280-015-2920-7 26811177 10.1007/s00280-015-2920-7 PMC4767869

[13] Badar T, HandisidesR DR, Benito JM, Richie MA, Borthakur G, Jabbour E, et al Phase I study of evofosfamide, an investigational hypoxia-activated prodrug, in patients with advanced leukemia. Am J Hematol. 2016;91: 800–805. doi: 10.1002/ajh.24415 27169385 10.1002/ajh.24415 PMC4946992

[14] Nakaichi M, Taura Y, Kanki M, Mamba K, Momoi Y, Tsujimoto H, et al Establishment and characterization of a new canine B-cell leukemia cell line. J Vet Med Sci. 1996;58: 469–471. 8741612 10.1292/jvms.58.469

[15] Momoi Y, Okai Y, Watari T, Goitsuka R, Tsujimoto H, Hasegawa A. Establishment and characterization of a canine T-lymphoblastoid cell line derived from malignant lymphoma. Vet Immunol Immunopathol. 1997;59: 11–20. 9437822 10.1016/s0165-2427(97)00053-6

[16] Zandvliet M, Teske E, Schrickx JA. Multi-drug resistance in a canine lymphoid cell line due to increased P-glycoprotein expression, a potential model for drug-resistant canine lymphoma. Toxicol In Vitro. 2014;28: 1498–1506. doi: 10.1016/j.tiv.2014.06.004 24975508 10.1016/j.tiv.2014.06.004

[17] Sia D, Alsinet C, Newell P, Villanueva A. VEGF signaling in cancer treatment. Curr Pharm. 2014;20: 2834–2842. 10.2174/13816128113199990590 23944367

[18] Appiah-Kubi K, Wang Y, Qian H, Wu M, Yao X, Wu Y. Platelet-derived growth factor receptor/platelet-derived growth factor (PDGFR/PDGF) system is a prognostic and treatment response biomarker with multifarious therapeutic targets in cancers. Tumour Biol. 2016; [Epub ahead of print] 10.1007/s13277-016-5069-z 27193823

[19] Shoeneman JK, Ehrhart EJ 3rd, Charles JB, Thamm DH. Survivin inhibition via EZN-3042 in canine lymphoma and osteosarcoma. Vet Comp Oncol. 2016; 14: e45–57. doi: 10.1111/vco.12104 24923332 10.1111/vco.12104

[20] Evens AM, Sehn LH, Farinha P, Nelson BP, Raji A, et al Hypoxia-inducible factor-1 {alpha} expression predicts superior survival in patients with diffuse large B-cell lymphoma treated with R-CHOP. J Clin Oncol. 2010;28: 1017–1024. doi: 10.1200/JCO.2009.24.1893 20048181 10.1200/JCO.2009.24.1893 PMC2834428

[21] Powell JR, Dojcinov S, King L, Wosniak S, Gerry S, Casbard A, et al Prognostic significance of hypoxia inducible factor-1α and vascular endothelial growth factor expression in patients with diffuse large B-cell lymphoma treated with rituximab. Leuk Lymphoma. 2013;54: 959–966. doi: 10.3109/10428194.2012.733875 23020605 10.3109/10428194.2012.733875

[22] Wolfesberger B, Arespacohaga A Guija de, Willmann M, Gerner W, Miller I, Schwendenwein I, et al Expression of vascular endothelial growth factor and its receptors in canine lymphoma. J Comp Pathol. 2007;137: 30–40. doi: 10.1016/j.jcpa.2007.03.003 17467003 10.1016/j.jcpa.2007.03.003

[23] Aricò A, Guadagnin E, Ferraresso S, Gelain ME, Iussich S, Rütgen BC. Platelet-derived growth factors and receptors in Canine Lymphoma. J Comp Pathol. 2014;151: 322–328. doi: 10.1016/j.jcpa.2014.07.001 25172054 10.1016/j.jcpa.2014.07.001

[24] Marconato L, Crispino G, Finotello R, Mazzotti S, Salerni F, Zini E. Serum lactate dehydrogenase activity in canine malignancies. Vet Comp Oncol. 2009;7: 236–243. doi: 10.1111/j.1476-5829.2009.00196.x 19891694 10.1111/j.1476-5829.2009.00196.x

[25] Sharif H, Euler H von, Westberg S, He E, Wang L, Eriksson S. A sensitive and kinetically defined radiochemical assay for canine and human serum thymidine kinase 1 (TK1) to monitor canine malignant lymphoma. Vet J. 2012;194: 40–47. doi: 10.1016/j.tvjl.2012.03.006 22516918 10.1016/j.tvjl.2012.03.006

[26] Greijer AE, Wall E van der. The role of hypoxia inducible factor 1 (HIF-1) in hypoxia induced apoptosis. J Clin Pathol. 2004;57: 1009–1014. doi: 10.1136/jcp.2003.015032 15452150 10.1136/jcp.2003.015032 PMC1770458

[27] Zandvliet M, Teske E, Schrickx JA, Mol JA. A longitudinal study of ABC transporter expression in canine multicentric lymphoma. Vet J. 2015; 205: 263–271. doi: 10.1016/j.tvjl.2014.11.002 25475167 10.1016/j.tvjl.2014.11.002

[28] Comerford KM, Wallace TJ, Karhausen J, Louis NA, Montalto MC, Colgan SP. Hypoxia-inducible factor-1-dependent regulation of the multidrug resistance (MDR1) gene. Cancer Res. 2002;62: 3387–3394. 12067980

[29] Hu J, Handisides DR, Valckenborgh Van E, Raeve DE H, Menu E, Broek Vande I, et al Targeting the multiple myeloma hypoxic niche with TH-302, a hypoxia-activated prodrug. Blood. 2010;116: 1524–1527. doi: 10.1182/blood-2010-02-269126 20530289 10.1182/blood-2010-02-269126

[30] Takahashi M, Goto-Koshino Y, Fukushima K, Kanemoto H, Nakashima K, Fujino Y, et al Phase I dose-escalation study of nimustine in tumor-bearing dogs. J Vet Med Sci. 2014;76: 895–899. doi: 10.1292/jvms.13-0345 24521794 10.1292/jvms.13-0345 PMC4108775

[31] Izaki S, Goto H, Okuda K, Matsuda M, Watanabe Y, Fujioka K. Long-term follow-up of busulfan, etoposide, and nimustine hydrochloride (ACNU) or melphalan as conditioning regimens for childhood acute leukemia and lymphoma. Int J Hematol. 2007;86: 253–260. doi: 10.1532/IJH97.06231 17988993 10.1532/IJH97.06231

[32] Pang YY, Wang T, Chen FY, Wu YL, Shao X, Xiao F, et al Glycolytic inhibitor 2-deoxy-d-glucose suppresses cell proliferation and enhances methylprednisolone sensitivity in non-Hodgkin lymphoma cells through down-regulation of HIF-1α and c-MYC. Leuk Lymphoma. 2015;56: 1821–1830. doi: 10.3109/10428194.2014.963575 25219592 10.3109/10428194.2014.963575

[33] Tuomela J, Grönroos TJ, Valta MP, Sandholm J, Schrey A, Seppänen J, et al Fast growth associated with aberrant vasculature and hypoxia in fibroblast growth factor 8b (FGF8b) over-expressing PC-3 prostate tumour xenografts. BMC Cancer. 2010;10: 596 doi: 10.1186/1471-2407-10-596 21034500 10.1186/1471-2407-10-596 PMC2984431

[34] Weiss GJ, Infante JR, Chiorean EG, Borad MJ, Bendell JC, Molina JR, et al Phase 1 study of the safety, tolerability, and pharmacokinetics of TH-302, a hypoxia-activated prodrug, in patients with advanced solid malignancies. Clin Cancer Res. 2011;17: 2997–3004. doi: 10.1158/1078-0432.CCR-10-3425 21415214 10.1158/1078-0432.CCR-10-3425

[35] Ganjoo KN, Cranmer LD, Butrynski JE, Rushing D, Adkins D, Okuno SH, et al A phase I study of the safety and pharmacokinetics of the hypoxia-activated prodrug TH-302 in combination with doxorubicin in patients with advanced soft tissue sarcoma. Oncology. 2011;80: 50–56. doi: 10.1159/000327739 21625179 10.1159/000327739

[36] Chawla SP, Cranmer LD, Tine Van BA, Reed DR, Okuno SH, Butrynski JE, et al Phase II study of the safety and antitumor activity of the hypoxia-activated prodrug TH-302 in combination with doxorubicin in patients with advanced soft tissue sarcoma. J Clin Oncol. 2014;32: 3299–3306. doi: 10.1200/JCO.2013.54.3660 25185097 10.1200/JCO.2013.54.3660 PMC4588714

