# Supplementary figures and images for: Hypoxia-activated prodrug TH-302 decreased survival rate of canine lymphoma cells under hypoxic condition
Source: PLoS One. 2017 May 10;12(5):e0177305. doi: 10.1371/journal.pone.0177305 (PMC5425042; doi:10.1371/journal.pone.0177305)

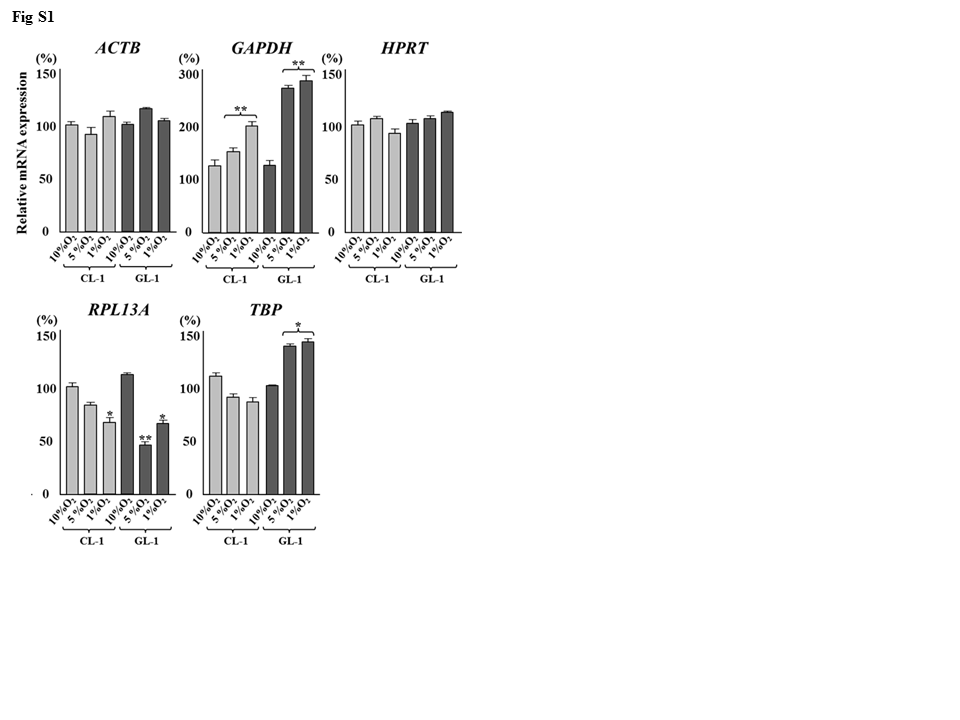

Supplement: S1 Fig — The mRNA expression, including β-actin (ACTB), glyceraldehyde-3-phosphate dehydrogenase (GAPDH), hypoxanthine phosphoribosyltransferase 1 (HPRT), ribosomal protein L13a (RPL13A), and TATA box-binding protein (TBP) were analyzed with qRT-PCR for selection of adequate internal reference gene. After 24 h in hypoxic culture (10%, 5% and 1% O2), the expression levels of these five genes in the CL-1 and GL-1 was quantified with the geNorm software (version 3.5). All expression was normalized to that of the control samples (21% O2), and each bar represents a mean ± SD. *P < 0.05 and **P < 0.01 (Dunnett’s test). (TIF) [file pone.0177305.s001.TIF]

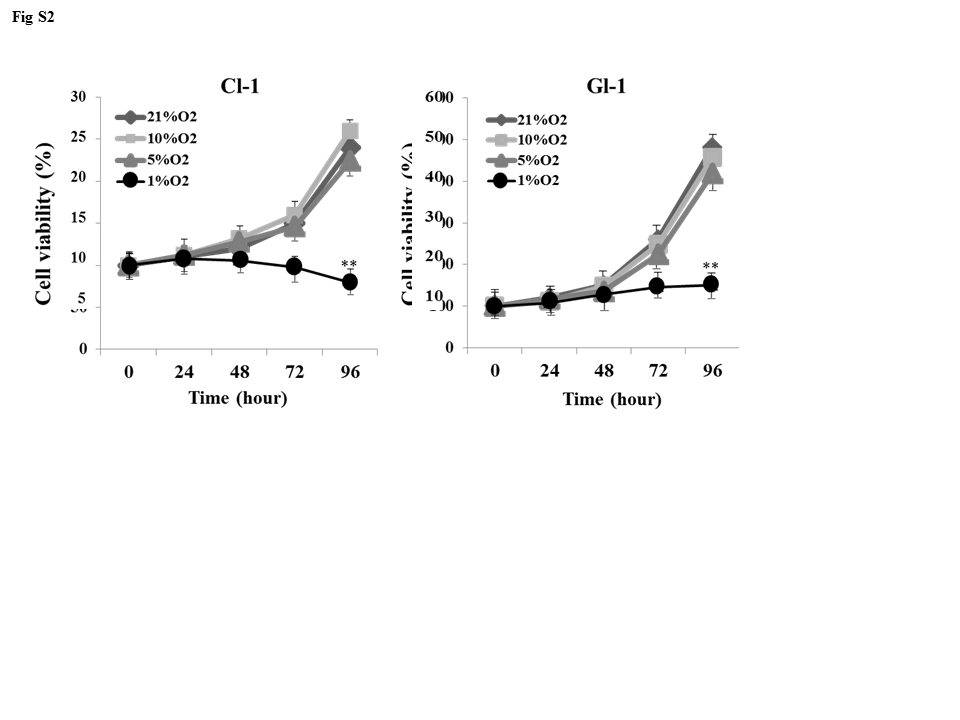

Supplement: S2 Fig — Relative cell viability is presented as a percentage (%) of the control value, and each bar represents a mean ± SD. **P < 0.01 vs the control cultured under 21% O2 (post hoc test). When cultured for 96 h under 1% O2, cell viability was significantly lower than that of the control cells (21% O2), whereas cell viability did not differ significantly under mildly hypoxic conditions (10% or 5% O2). (TIF) [file pone.0177305.s002.TIF]

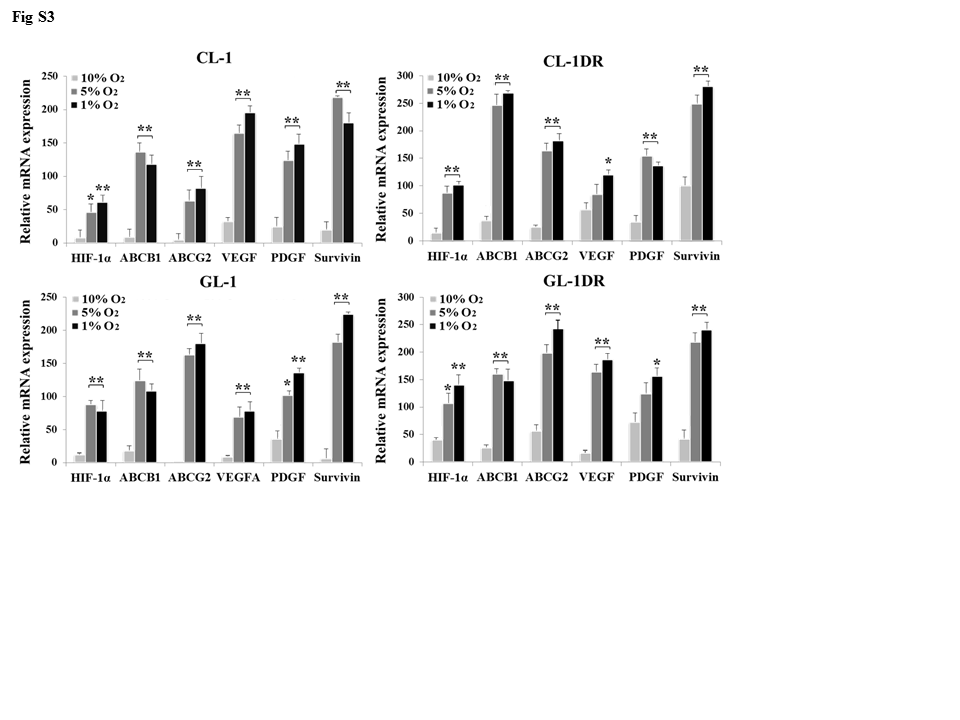

Supplement: S3 Fig — After 24 h in hypoxic culture (10%, 5%, or 1% O2), the mRNA expression of hypoxia-inducible factor 1α (HIF-1α), ATP-binding cassette transporter B1 (ABCB1), ATP-binding cassette transporter G2 (ABCG2), endothelial growth factor (VEGF), platelet-derived growth factor (PDGF), and survivin was analyzed with qRT-PCR. All expression was normalized to that of the control samples (21% O2), and each bar represents a mean ± SD. *P < 0.05 and **P < 0.01 (Dunnett’s test). (TIF) [file pone.0177305.s003.TIF]

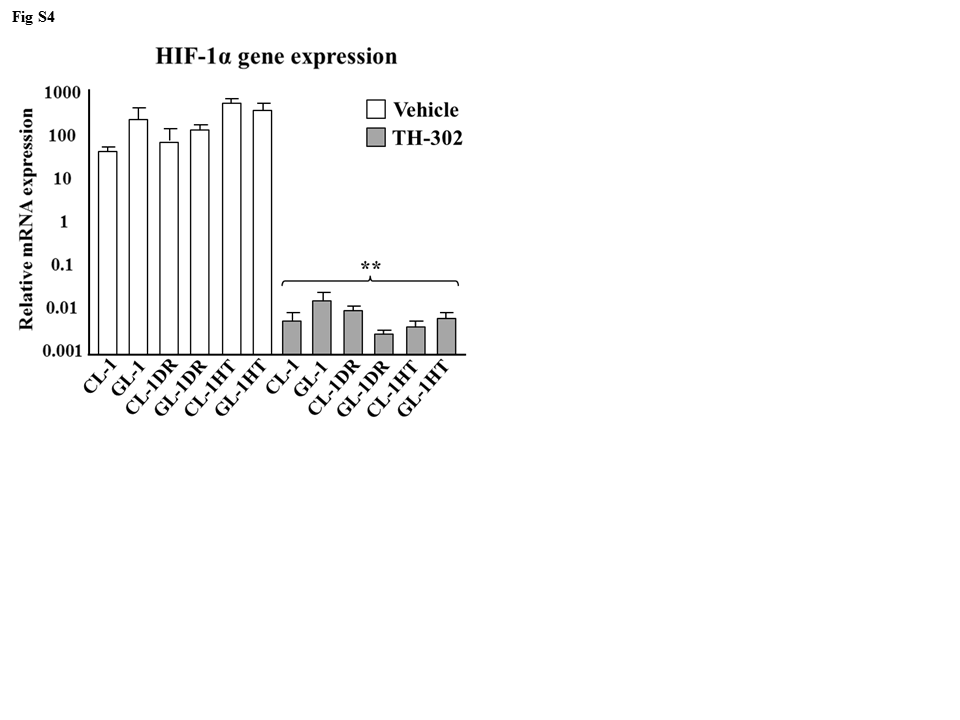

Supplement: S4 Fig — After treatment with 50 μM TH-302 for 12 h during culture under 5% O2, the mRNA expression was analyzed with qRT-PCR. All expression was normalized to that of the control samples (21% O2), and each bar represents a mean ± SD. *P < 0.05 and **P < 0.01 (Dunnett’s test). (TIF) [file pone.0177305.s004.TIF]

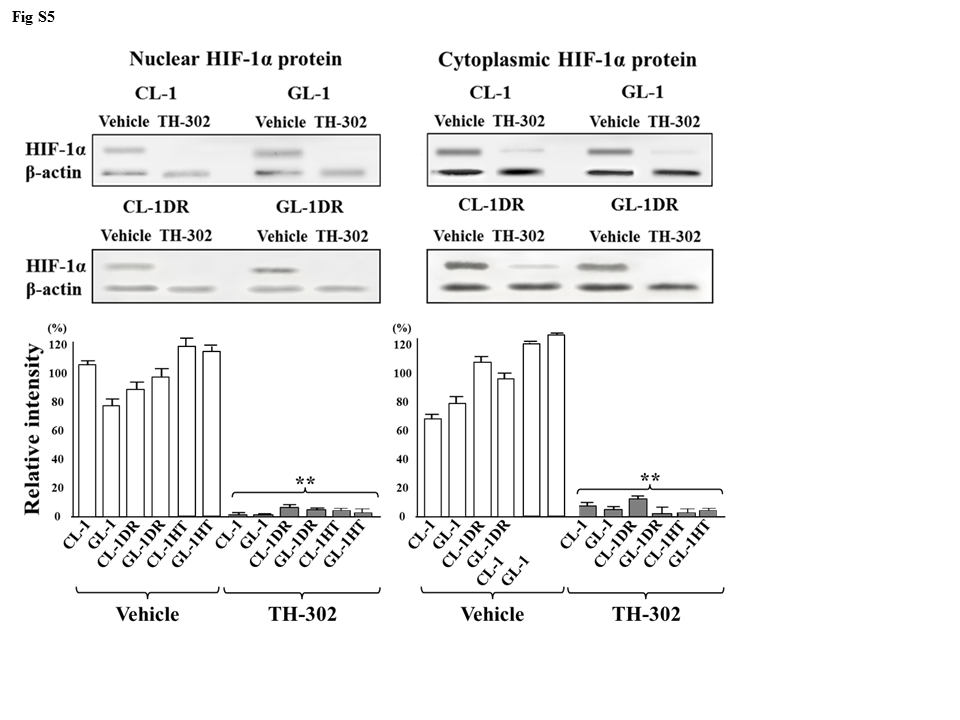

Supplement: S5 Fig — After treatment with 50 μM TH-302 for 12 h during culture under 5% O2, the nuclear and cytosolic localization of HIF-1α was detected with western blotting. Immunoreactive band intensities are presented as percentages (%) of the control values. Each bar represents the mean ± SD of three separate experiments. **P < 0.01 vs control (Dunnett’s test). (TIF) [file pone.0177305.s005.TIF]
