# Supplementary material for: Hypoxia-activated prodrug TH-302 decreased survival rate of canine lymphoma cells under hypoxic condition
Source: PLoS One. 2017 May 10;12(5):e0177305. doi: 10.1371/journal.pone.0177305 (PMC5425042; doi:10.1371/journal.pone.0177305)
Supplement: S1 Table — (DOCX) [file pone.0177305.s006.docx]

**Supporting Tables**

**S1 Table**: Baseline information on the canine lymphoma cells

| Cell line | IC_50_ of DOX (nM) ^a)^ | mRNA expression ^b)^ | |
| --- | --- | --- | --- |
|  |  | *ABCB1* | *ABCG2* |
| CL-1 | 5.8 | 1.0 | 1.0 |
| GL-1 | 10.4 | 1.82 | 0.8 |
| CL-1DR | 58.0 | 64.3 | 96.2 |
| GL-1DR | 124.6 | 145.6 | 62.4 |

a) 50% inhibitory concentration (IC_50_) of doxorubicin (DOX) was evaluated with an MTT assay.

b) Levels of mRNA expression in cells were evaluated with real-time PCR and normalized to those of the same mRNA in CL-1 cells.

*ABCB1*: *ATP-binding cassette transporter B1*

*ABCG2*: *ATP-binding cassette transporter G2*
