# Supplementary material for: Hypoxia-activated prodrug TH-302 decreased survival rate of canine lymphoma cells under hypoxic condition
Source: PLoS One. 2017 May 10;12(5):e0177305. doi: 10.1371/journal.pone.0177305 (PMC5425042; doi:10.1371/journal.pone.0177305)
Supplement: S2 Table — (DOCX) [file pone.0177305.s007.docx]

**Supporting table 2**

**S2 Table**: Target primer sequences used in this study

| Target gene | Sequences | | Amplicon size  (bp) | | GenBank |
| --- | --- | --- | --- | --- | --- |
| HPRT1 | Forward | 5′-TGCTCGAGATGTGATGAAGG-3′ | | 192 | NM001003357 |
|  | Reverse | 5′-TCCCCTGTTGACTGGTCATT-3′ | |  |  |
| HIF-1α | \| Forward 5′-TTACGTTCCTTCGATCAGTTGTA-3′  Reverse 5′-GAGGAGGTTCTTGCATTGGAGTC-3′ \| \| \| \|  \| \| --- \| --- \| --- \| --- \| --- \| \|  \| \|  \| \| \| \|  \| \|  \| \| \| \|  \| \|  \| \| \| \| Reverse \| \| 5′-TCCCCTGTTGACTGGTCATT-3′ \| \| \| | | | 105 | AY455802 |
| ABCB1 | Forward | 5′-CTATGCCAAAGCCAAAGTATC-3′ | | 80 | NM001003215 |
|  | Reverse | 5′-GAGGGCTGTAGCTGTCAATC-3′ | |  |  |
| ABCG2 | Forward | 5′-GGTATCCATAGCAACTCTCCTCA-3′ | | 146 | NM001048021 |
|  | Reverse | 5′-GCAAAGCCGCATAACCAT-3′ | |  |  |
| PDGFα | Forward | 5′-TTTGGAAGCAAGTCTGAGAGCC-3′ | | 100 | NM001190172.1 |
|  | Reverse | 5′-TGGCCTCCTCAATGCTTCTT-3′ | |  |  |
| VEGF-A | Forward | 5′-TTGCTGCTCTACCTCCACCAT-3′ | | 64 | NM001003175.2 |
|  | Reverse | 5′-TGTGCTCTCCTCCTGCCATAG-3′ | |  |  |
| Survivin | Forward | 5′-TCGAAGAGACCGCAAAGAAAGTGC-3′ | | 181 | AY741504 |
|  | Reverse | 5′-GAATTGTGGCCGTTCTCCTTTCCT-3′ | |  |  |
