# Supplementary material for: Hypoxia-activated prodrug TH-302 decreased survival rate of canine lymphoma cells under hypoxic condition
Source: PLoS One. 2017 May 10;12(5):e0177305. doi: 10.1371/journal.pone.0177305 (PMC5425042; doi:10.1371/journal.pone.0177305)
Supplement: S1 File — (DOCX) [file pone.0177305.s008.docx]

**Supporting Methods**

**Establishment of doxorubicin (DOX)-resistant canine lymphoma cell lines**

CL-1DR and GL-1DR cells were established as DOX-resistant lymphoma cells as follows. Briefly, CL-1 and GL-1 cells were cultured in RPMI 1640 (Gibco, Grand Island, NY, USA) supplemented with 10% heat-inactivated fetal bovine serum (Cosmo Bio, Tokyo, Japan), 1% L-glutamine (BioWhittaker, Walkersville, MD, USA), 100 U/mL penicillin, 100 µg/ml streptomycin, and 10 ng/mL DOX (Sigma, St Louis, MO, USA) at 37 °C under 5% CO_2_, and the concentration of DOX in the medium was doubled every 2 months. After culture for 6 months in medium containing DOX (final concentration 0.07 µM, 40 ng/mL), the enhanced DOX resistance of the CL-1 and GL-1 cells was confirmed with two different methods. The 50% inhibitory concentration (IC_50_) of DOX for the cells was calculated relative to the control cells with a Cell Proliferation Kit I according to the manufacturer’s instructions (Roche, Indianapolis, IN, USA). The cells were treated with various concentrations of DOX (1–1,000 µM) in 96-well plates. After the cells were treated with DOX for 24 h, 10 µL of the 3-(4,5-dimethylthiazol-2-yl)-2,5-diphenyltetrazolium bromide (MTT) solution was added to each well and the cells were maintained in an incubator for 4 h under 5% CO_2_ at 37 °C. The supernatants were removed from the wells and replaced with 100 µL of the solubilization solution. After the plates were shaken, the absorbance of the solution in each well was measured at 570 nm using an iMark microplate spectrophotometer (Bio-Rad, Hercules, CA, USA). The IC_50_ values are presented as the medians of triplicate samples, and the experiments were repeated three times (S1 Table). We also used qRT-PCR (as described in the next subsection) to measure the mRNA expression of the multidrug-resistance genes ATP-binding cassette transporter B1 (ABCB1) and ATP-binding cassette transporter G2 (ABCG2) (S1 Table). Based on these results, sublines of the CL-1 and GL-1 cells with low sensitivity to DOX were selected and used as the CL-1DR and GL-1DR cells, respectively, in this study.

**Quantitative real-time reverse transcription polymerase chain reaction (qRT-PCR)**

After culture for 24 h under normoxic (21% O_2_; control) or hypoxic conditions (10%, 5%, and 1% O_2_), the expression of *HIF-1α*, *ABCB1*, *ABCG2*, *PDGF*, *VEGF*, and *survivin* in the cells was evaluated with qRT-PCR. Additionally, after treatment with 50 µM TH-302 for 12 h during culture under 21% and 5% O_2_, the mRNA expression of *HIF-1α* was analyzed. Total RNA was isolated from 1 × 10^6^ cells with an mirVana miRNA Isolation Kit (Ambion, Thermo Fisher Scientific, Waltham, MA, USA). Total RNA was assessed with a NanoDrop 2000 spectrophotometer (Thermo Fisher Scientific) and the quality of the isolated RNA (RNA integrity number: RIN ≥ 8.6) was determined with the Bioanalyzer System (Agilent Technologies, Waldbroon, Germany). Total RNA (≥ 100 ng/mL) was reverse transcribed into cDNA using ReverTra Ace qPCR RT Master Mix with gDNA Remover (Toyobo, Osaka, Japan). Candidate internal reference genes were selected for quantitative analysis, including β-actin (*ACTB*), glyceraldehyde-3-phosphate dehydrogenase (*GAPDH*), hypoxanthine phosphoribosyltransferase 1 (*HPRT*), ribosomal protein L13a (*RPL13A*), and TATA box-binding protein (*TBP*). To normalize the amounts of cDNA in the samples used, the expression of these five genes was quantified, and the accuracy of the internal control was analyzed with the geNorm software (version 3.5). The *HPRT* gene was subsequently used as the internal reference based on the results of this analysis (S1 Fig). All target primer sequences used in this study were designed with the Primer3 interface (http://frodo.wi.mit.edu/) or have been reported previously (Table S2). The qRT-PCR assays were performed with the StepOnePlus™ Real-Time PCR System (Applied Biosystems; Thermo Fisher Scientific) and SYBR Premix Ex Taq II (Tli RNaseH Plus; Takara, Shiga, Japan). The thermal cycling conditions included an initial denaturation step at 95 °C for 3 min and then 40 amplification cycles (95 °C for 20 s, 60 °C for 20 s). Relative mRNA expression was normalized to the control samples (21% O_2_) using the 2^−ΔΔCt^ method and is presented as relative quantitative values.

**Western blotting**

After the cells were cultured for 12 h under normoxia (21% O_2_) or hypoxia (5% or 1% O_2_) and then treated for 12 h with 50 µM TH-302 under 21% or 5% O_2_, the total, nuclear and cytoplasmic proteins were extracted from them with the Nuclear/Cytosolic Fraction Kit (Cell Biolabs, San Diego, CA, USA), according to the manufacturer’s instructions. Briefly, the cells were homogenized in ice-cold cytosol extraction buffer and cell lysis reagent was added. The homogenate was centrifuged at 800 × g, and the supernatant was separated and saved as the cytosolic fraction. The nuclear fraction was washed several times with nuclear extraction buffer and incubated on ice for 30 min. The mixture was centrifuged at 14,000 × g for 30 min. The supernatant was separated and stored at −80 °C until analysis. Anti-α-tubulin and anti-lamin-A + C antibodies were used to confirm the cytosolic and nuclear fractions, respectively.

The cells were washed twice with ice-cold Tris-buffered saline (TBS; Sigma) and then lysed with 100 µL of radio-immunoprecipitation assay (RIPA) buffer (Sigma). The protein concentrations were determined with the Bradford protein assay using bovine serum albumin (BSA; Sigma) as the standard, and 20 µg of protein from each sample was loaded onto a gel for electrophoresis. The proteins were denatured, subjected to sodium dodecyl sulfate polyacrylamide gel electrophoresis (10% gels; Sigma), and electrotransferred onto nitrocellulose membranes (Whatman, Piscataway, NJ, USA) in a semidry transfer apparatus (Bio Craft, Tokyo, Japan). The membranes were incubated for 1 h at room temperature in a blocking solution: 10 mM Tris-HCl (pH 7.4), 0.15 M NaCl, 0.1% Tween 20, 1% BSA, and 0.05% NaN_3_. The membranes were then incubated overnight at 4 °C with the primary antibodies, including a mouse monoclonal antibody directed against β-actin (G043, diluted 1:1,000; Abcam, Cambridge, MA, USA) and a rabbit polyclonal anti-HIF-1α antibody (NB100-449, diluted 1:500; Littleton, CO, USA). The membranes were washed three times for 5 min each with Tween-TBS (10 mM Tris-HCl, 0.15 M NaCl, and 0.1% Tween 20) and then incubated with a horseradish-peroxidase-conjugated anti-rabbit-IgG secondary antibody (diluted 1:1,000; Fischer Scientific Thermo, Pittsburgh, PA, USA) in Tween-TBS for 1 h at room temperature. The immunoreactive bands were visualized with a chemiluminescence system (Ez-Capture MG, Atto, Tokyo, Japan), using Amersham ECL Prime Western Blotting Detection Reagent (GE Healthcare, Princeton, NJ, USA). The bands of HIF-1α on the western blots were quantified with the ImageJ software (version 1.451: http://imagej.nih.gov/ij/) and are presented as relative intensities normalized to that of β-actin.
